# ROS-responsive dimeric prodrug-based nanomedicine targeted therapy for gastric cancer

**DOI:** 10.1080/10717544.2021.1937380

**Published:** 2021-06-18

**Authors:** Jiachi Ma, Yuzhong Chen, Wanqing Liang, Lei Li, Jun Du, Chengwu Pan, Chensong Zhang

**Affiliations:** a Department of Oncological Surgery, The First Affiliated Hospital of Bengbu Medical College, Bengbu, People's Republic of China; b Bengbu Medical College, Bengbu, People's Republic of China

**Keywords:** ROS-responsive, dimeric prodrug, active targeting, high drug loading

## Abstract

Gastric cancer (GC) remains a major public health problem. Ursolic acid (UA) is reported to be effective in inhibiting GC; however, its low solubility and poor biocompatibility have greatly hindered its clinical application. Herein, an innovative reactive oxygen species (ROS)-sensitive UA dimeric prodrug is developed by coupling two UA molecules via a ROS-cleavable linkage, which can self-assemble into stable nanoparticles in the presence of surfactant. This new UA-based delivery system comprises the following major components: (I) dimeric prodrug inner core that can achieve high drug-loading (55%, w/w) and undergo rapid and selective conversion into intact drug molecules in response to ROS; (II) a polyethylene glycol (PEG) shell to improve colloid stability and extend blood circulation, and (III) surface-modified internalizing RGD (iRGD) to increase tumor targeting. Enhancement of the antitumor effect of this delivery system was demonstrated against GC tumors *in vitro* and *in vivo*. This novel approach offers the potential for clinical applications of UA.

## 1. Introduction

Gastric cancer (GC) is the fifth most frequent cause of cancer-related mortality around the world (Bray et al., 2018). As the early clinical symptoms are not obvious, most patients with GC are always diagnosed late (Zhang et al., 2019). Patients with late-stage GC usually show poor outcomes, with an overall five-year survival rate of less than 20% (Guo et al., 2019). Currently, chemotherapy is the main therapeutic strategy for late-stage GC patients. However, the drawbacks of traditional chemotherapy drugs including low water solubility and poor biocompatibility result in poor efficacy (Xu et al., 2019; Zhang et al., 2019).

In recent years, the application of traditional Chinese medicine to cancer therapy has attracted increased attention. Ursolic acid (UA), a typical pentacyclic triterpene acid, is widespread in various plants, including *Salvia officinalis*, *Sanguisorba officinalis*, and *Rosmarinus officinalis* (Shao et al., 2020). Multiple pharmacological properties of UA, such as its anti-tumor, antioxidant, anti-inflammatory, and antimicrobial effects, have been confirmed by various studies (Ji et al., 2018; Shen et al., 2018). Recently, UA has attracted great attention for its remarkable cancer-growth-inhibiting properties with minimal side effects (Liu et al., 2017). Previous studies have shown that UA can effectively suppress the growth of various tumors, including hepatocellular carcinoma, breast cancer, GC, and colon cancer, both *in vitro* and *in vivo* (Kim and Moon, 2015; Jaman and Sayeed, 2018; Shen et al., 2018; Ou et al., 2020). However, insolubility, short blood circulation times, and low bioavailability have greatly limited the application of UA in the clinic (Liu et al., 2018). To overcome these bottlenecks, many UA-based drug delivery methods have been developed in recent years (Liu et al., 2017; Shen et al., 2018). For instance, Jiang et al. developed a mesoporous silica nanoparticle (MSN)-based nanosystem for UA that could effectively deliver UA to HeLa cancer cells (Yuan et al., 2012). Further, Ji et al. reported a redox-responsive UA prodrug delivery system that could effectively inhibit the growth and migration of MCF-7 cells (Ji et al., 2018). However, several limitations that hinder the translation of UA delivery systems to the clinic remain unresolved, especially low drug loading, poor tumor selectivity, and insufficient release of the active parent drugs in cancer cells (Li et al., 2020a,b,c).

Recently, dimeric prodrugs-based nanosystems (DPNS) have attracted considerable attention (Van der Meel et al., 2019). In these systems, dimeric prodrugs are prepared by connecting two drug molecules with appropriate linkages, and that can be converted into the active parent drug *in vivo* (Sun et al., 2018). Dimeric prodrugs can undergo self-assembly into nanoparticles with or without surfactants in an aqueous solution to form the DPNS; therefore, DPNS can achieve high drug-loading efficiency, usually more than 50% (Jiang et al., 2017; Li et al., 2020a). Moreover, for effective conversion of dimeric prodrugs to bioactive parent drugs in cancer cells, various stimuli-sensitive linkages, such as disulfide bonds, diselenide bonds, azobenzene bonds, and the thioketal (TK) moiety have used to prepared DPNS, which can release the active parent drug in response to high levels of glutathione (GSH), reactive oxygen species (ROS), and hypoxic conditions (Luo et al., 2014; Song et al., 2016; Pei et al., 2018; Li et al., 2019, 2020a,b,c; Zhou et al., 2020; Zuo et al., 2020). It is reported that ROS-responsive drug delivery systems exhibit higher selectively during drug release in tumor cells than GSH- and acidic pH-sensitive drug delivery systems, because both high concentrations of GSH and lysosomes (with acidic pH) are present in both normal and cancer cells (Ye et al., 2017). The concentration of ROS in cancer cells is about 10-fold higher than in normal cells (Ye et al., 2017; Tao and He, 2018). Thus, ROS-based drug delivery systems may enable the selective and rapid release of drugs in tumor cells.

Additionally, drug delivery systems with active targeting to tumors are an effective strategy to enhance tumor selectivity (Li et al., 2017). Various target ligands, including folic acid, peptides, and antibodies, have been developed for targeted drug delivery (Zhao et al., 2020). Among them, internalizing RGD (iRGD) peptide, a peptide that specifically binds to αvβ3 integrins, has been widely used in tumor-targeted drug delivery (Wang et al., 2014; Simon-Gracia et al., 2016; Zhang et al., 2016). αvβ3 integrin, a transmembrane protein, is overexpressed in various tumor cells (e.g. GC cancer) and expressed at low levels in normal cells (Yang et al., 2018; Mahmoudi Saber, 2019; Shi et al., 2019). Therefore, DPNS modified with iRGD may significantly improve tumor-targeting ability.

**Scheme 1.**
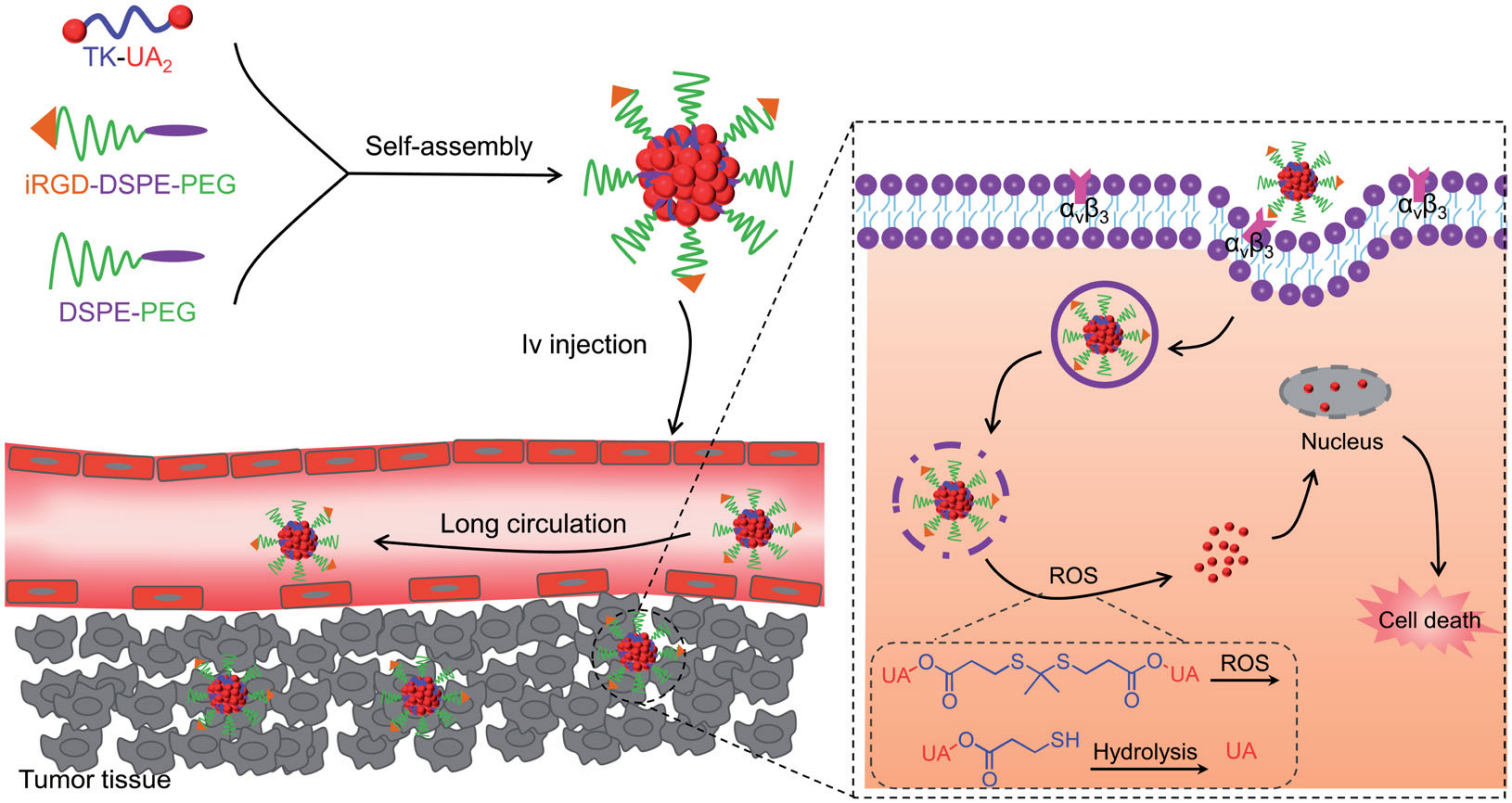
Illustration of the UA-based DPNS and its active targeting to GC and intracellular ROS-triggered drug release.

Here, we designed and developed an ROS-responsive and iRGD-encoded UA-based DPNS for GC-targeted therapy (Scheme 1). The UA-based dimeric prodrug was prepared by conjugating two UA molecules through a TK linker (TK-UA_2_). Then, UA-based DPNS was prepared, characterized, and evaluated in human SGC 7901 cells and SGC 7901 tumor-bearing mice in detail.

## 2. Experimental

### 2.1. Materials

1,2-Distearoyl-sn-glycero-3-phosphoethanolamine-N-[methoxy(polyethylene glycol)-2000] (DSPE-PEG) and 1,2-distearoyl-sn-glycero-3-phosphoethanolamine-N-[iRGD-(polyethylene glycol)-2000] (DSPE-PEG-iRGD) were obtained from Xi’an Ruixi Biological Technology Co., Ltd. (Xi’an, China). 3-Mercaptopropionic acid, acetone, anhydrous *N*,*N*-dimethylformamide (DMF), 1-(3-dimethylaminopropyl)-3-ethylcarbodiimide hydrochloride (EDC), 4-dimethylaminopyridine (DMAP), and UA were purchased from Aladdin Reagent Co., Ltd. (Shanghai, China). 3-[4,5-Dimethylthiazol-2-yl-]-2,5-diphenyltetrazoliumbromide (MTT), RPMI 1640 medium, and DAPI were purchased from Beyotime Biotechnology (Shanghai, China).

#### 2.1.1. Cell and animals

Human GC cell lines SGC 7901 and NIH-3T3 cells were obtained from KeyGEN BioTECH (Nanjing, China) and cultured using Dulbecco's modified Eagle's medium (DMEM) supplemented with 10% fetal bovine serum (FBS), 100 U/mL penicillin, and 100 μg/mL streptomycin in a humidified incubator containing 5% CO_2_ at 37 °C.

Male BALB/c nude mice were supplied by the Bengbu Medical University Experimental Animal Center (Bengbu, China). All *in vivo* procedures were performed in adherence to the guidelines of the Institutional Animal Care and Use and the study was approved by the Ethics Committee of the Bengbu Medical University.

### 2.2. TK fabrication

TK was synthesized as previously reported (Yin et al., 2019; Chang et al., 2020). Briefly, acetone and 3-mercaptopionic acid were mixed at a 9:5 molar ratio and stirred at room temperature under a dry hydrogen chloride atmosphere for 6 h. After the reaction, the mixture was crystallized under an ice-salt mixture environment. Then, the mixture was filtered to obtain crystals, which were purified by washing with hexane and cold water many times, and then dried under vacuum to obtain TK (yield: 41.2%). TK was confirmed by proton nuclear magnetic resonance spectroscopy (^1^H NMR AVANCE III, BRUKER, Fällanden, Switzerland) and mass spectrometry (MS, AB SCIEX 6500 Qtrap, Applied Biology, Inc., Irvine, CA).

### 2.3. TK-UA_2_ fabrication

In brief, UA (10.05 g, 0.022 mol), TK (2.51 g, 0.01 mol), EDC (8.40 g, 0.044 mol), and DMAP (3.90 g, 0.032 mol) were added to the solution of in 20.0 mL of anhydrous DMF. The mixture was stirred under an N_2_ environment at room temperature for 1 h. Subsequently, additional EDC (8.40 g, 0.044 mol) and DMAP (3.90 g, 0.032 mol) were added to the above mixture and reacted for a further 24 h at 20 °C in N_2_. Then, the mixture was concentrated *in vacuo* to remove the solution. The obtained crude product was purified by silica column chromatography using ethyl acetate/hexane = 4:1 as the mobile phase. Finally, TK-UA_2_ was obtained with a yield of 67.3%.

As a control, the ROS-insensitive dimeric prodrug, CC-UA_2_, was also prepared by conjugating UA to adipic acid using the same method. The yield of CC-UA_2_ was 71.3%. TK-UA_2_ and CC-UA_2_ were characterized by ^1^H NMR and MS.

### 2.4. Preparation of drug-loading nanoparticles

The NPs formed by TK-UA_2_, DSPE-PEG-iRGD, and DSPE-PEG were denoted as iRGD-TK-NPs; NPs formed by TK-UA_2_ and DSPE-PEG were named as TK-NPs, NPs formed by CC-UA_2_, DSPE-PEG-iRGD, and DSPE-PEG were dubbed as iRGD-CC-NPs, and NPs formed by CC-UA_2_ and DSPE-PEG were defined as CC-NPs. The ethanol injection method was used to prepare these NPs. Typically, 8.0 mg TK-UA_2_, 1.0 mg DSPE-PEG-iRGD, and 2.0 mg DSPE-PEG were dissolved in 4.0 mL of ethanol. Subsequently, the mixture was added dropwise to 10.0 mL distilled water under vigorous stirring. After stirring for 2 h, ethanol was removed by evaporating at 25 °C under vacuum. Then, the colloidal solution was diluted to 10.0 mL with distilled water to obtain the iRGD-TK-NPs. The zeta potential and hydrodynamic diameter of NPs were determined by dynamic light scattering (DLS) on a Zetasizer (Zs90, Malvern, Malvern, UK). Additionally, coumarin-6 loaded NPs were also prepared by the same operating procedures.

### 2.5. Stability assay

NPs were cultured at 37 °C in PBS containing 10% FBS. At prescriptive intervals time points, the size of NPs was measured by DLS.

### 2.6. Evaluation of ROS-responsive ability

Changes in size and *in vitro* drug release assay were performed to investigate the ROS-responsive ability of NPs. For size changes assay, iRGD-TK-NPs and iRGD-CC-NPs were incubated in PBS containing 10 mM H_2_O_2_ at 37 °C. After incubation for 2, 4, 8, 12, and 24 h, the size of NPs was measured by DLS.

For drug release analysis, PBS (pH 7.4) containing various concentrations of H_2_O_2_ (1.0, 0.1, and 0 mM) was used as the release medium. A quantitative amount of iRGD-TK-NPs was added to the release medium and incubated at 37 °C with gentle stirring. At prescriptive intervals, the amount of UA released was measured by high-performance liquid chromatography (HPLC) method (mobile phase: methanol/water/trifluoroacetate (80:20:0.05, v/v), 1 mL/min, UV–vis detector set at 210 nm according to full wavelength scan using a Shimadzu HPLC system (LC20A, Tokyo, Japan).

### 2.7. Cellular uptake investigation

Confocal laser scanning microscopy (CLSM) and flow cytometry (FCM) assay were performed to investigate the cell internalization. For the CLSM study, 10,000 SGC 7901 cells were seeded into a 35-mm glass-bottom culture dish and cultured in medium overnight at 37 °C. After incubation, coumarin-6 loaded iRGD-TK-NPs and TK-NPs solution diluted with FBS-free DMEM at a final concentration of 500 ng/mL (equal to coumarin-6) was added, and incubated for a further 1 h or 3 h. After treatment, cells were washed with PBS, stained with DAPI, fixed in 4% paraformaldehyde, and then observed by CLSM (LSM 780, Zeiss, Jena, Germany).

For FCM assay, SGC 7901 and NIH-3T3 cells were seeded into a six-well plate at a density of ten thousand cells per well, respectively, and cultured overnight at 37 °C. After incubation, coumarin-6 loaded NPs solution diluted with FBS-free DMEM at a final concentration of 500 ng/mL (equal to coumarin-6) were added and incubated for 1, 2, 3, or 4 h. After treatment, cells were washed with PBS and detected by FCM (BD FACSCalibur, Aria III, Piscataway, NJ).

### 2.8. *In vitro* cytotoxicity

SGC 7901 and NIH-3T3 cells were seeded in 96-well plates at a density of 3000 cells per well and incubated for 24 h. Subsequently, cells were treated with UA, iRGD-TK-NPs, TK-NPs, CC-NPs, or iRGD-CC-NPs at various concentrations for 48 h at 37 °C. After treatment, 20 μL of MTT solution was added into each well and cultured for a further 4 h. At the end of the incubation period, the medium was removed and 200 μL of DMSO was added; then, the absorbance was determined using a microplate reader (Bio-Rad, Hercules, CA). Cell viability was calculated according to the following equation:

$$\text{cell}\;\text{viability}\;=\;\left({\text{OD}}_{\text{treatment}}/{\text{OD}}_{\text{control}}\right)\;\times \;100\%.$$





### 2.9. Hemolysis assay

Sprague-Dawley (SD) rat blood was obtained and washed with saline. Then, RBCs were collected and mixed with NPs solution at various concentrations. The mixture was maintained at 37 °C with slight stirring for 2 h. Subsequently, the absorbance was detected using a microplate reader at 577 nm. Saline and Triton X-100 were employed as negative and positive controls. The following equation was used to calculate hemolysis:

$$\text{Hemolysis}\;\left(\%\right)=\left({\text{OD}}_{\text{sample}}-{\;\text{OD}}_{\text{negative}}\right)/\left({\text{OD}}_{\text{positive}}\;–{\;\text{OD}}_{\text{negative}}\right)\times 100\%.$$





### 2.10. Pharmacokinetics and biodistribution

The SD rat was used as the animal model to investigate the *in vivo* pharmacokinetics of all drug formulations. UA, iRGD-TK-NPs, TK-NPs, and iRGD-CC-NPs were injected into SD rats via the tail vein at a UA-equivalent concentration of 11.0 mg/kg, respectively. At pre-set time points, blood samples were obtained from the orbital plexus and centrifuged at 3000×*g* for 10 min. The supernatant was collected and mixed with methanol/chloroform (1:2, v/v) to extract the UA prodrug and free UA. After centrifugation, the supernatant was collected, dried under vacuum, redissolved with methanol, and measured by HPLC as described above.

The SGC 7901 xenograft tumor mouse was used as the animal model to study the biodistribution of all drug formations. SGC 7901 tumor-bearing mice were prepared by subcutaneous inoculation of 6.0 × 10^6^ cells into the hind flank of each mouse. Two weeks after injection of cells, mice were intravenously injected with UA, iRGD-TK-NPs, TK-NPs, and iRGD-CC-NPs at the UA-equivalent concentration of 11.0 mg/kg. After treatment for 6 h and 12 h, six mice were euthanized by cervical dislocation; then, the major organs (kidney, spleen, liver, heart, and lung) and tumor tissues were collected, washed with PBS, weighed, and detected by HPLC.

### 2.11. *In vivo* antitumor effect

SGC 7901 xenograft tumor mice were randomly divided into four groups when the tumor volume reached about 80 mm^3^ and then treated with saline, UA, iRGD-TK-UA, TK-NPs, and iRGD-CC-UA, respectively, at UA-equivalent 11.0 mg/kg five times every three days. After the first administration, the tumor’s length/width and mouse body weight were measured every three days. The following equation was used to calculate the tumor volume: Volume = 1/2 × length×(width)^2^. After treatment for 21 days, mice were sacrificed and tumor tissues were collected. The tumors were imaged, weighed, and tumor growth suppression (TGS) was calculated as follows:

$$\text{TGS}\;\left(\%\right)=\left({\text{weight}}_{\text{saline}}–{\;\text{weight}}_{\text{treatment}}\right)/{\text{weight}}_{\text{saline}}\times 100\%.$$





## 3. Results and discussion

### 3.1. TK-UA_2_ characterization

**Figure 1.**
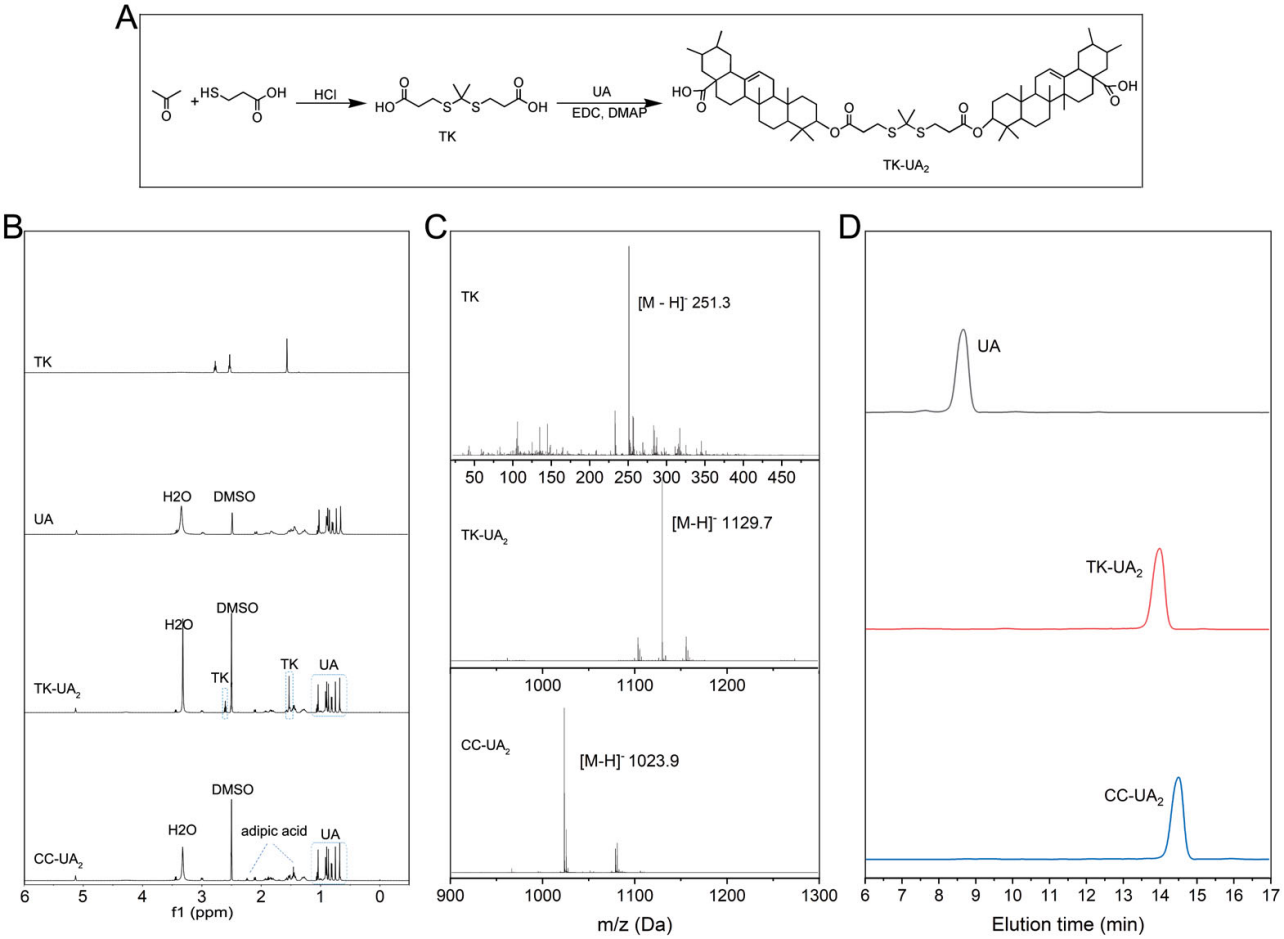
Characterization of TK, TK-UA_2_, and CC-UA_2_. (A) ^1^H NMR spectrum of TK, TK-UA_2_, and CC-UA_2_, respectively. (B) MS spectrum of TK, TK-UA_2_, and CC-UA_2_, respectively. (C) HPLC spectrum of UA, TK-UA_2_, and CC-UA_2_, respectively.

As exhibited in Figure 1(A), TK-UA_2_ was synthesized through two steps: the ROS-sensitive linkage, TK, was fabricated; subsequently, UA was conjugated to TK to obtain TK-UA_2_. ^1^H NMR and MS were used to characterize the TK and TK-UA_2_. ^1^H NMR results are presented in Figure 1(B); all peaks were well assigned. In the TK spectrum, the signal at 2.3 ppm and 1.4 ppm was attributed to the methyl group and the signal at 4.3 ppm to the methylene. The peaks of TK were consistent with those described in previous reports, demonstrating that TK was successfully prepared. Moreover, MS results showed that the molecular weight of TK was 253.3 Da (Figure 1(C)), which was consistent with the theoretically calculated value, further confirming the successful synthesis of TK. In the ^1^H NMR spectrum of TK-UA (Figure 1(D)), both the characteristic peaks of UA (2.3 ppm and 5.6 ppm) and TK were observed, suggesting successful preparation of TK-UA_2_. MS results also demonstrated that TK-UA_2_ was successfully prepared. As a control, CC-TK was constructed and characterized by ^1^H NMR and MS (Figure 1(C)). Both ^1^H NMR and MS results confirmed that CC-UA_2_ was also successfully prepared (Figure 1(B,C)). Additionally, HPLC was performed to evaluate the purity of TK-UA_2_ and CC-UA_2_. As shown in Figure 1(D), no peaks corresponding to free UA appeared in either the spectrum of TK-UA_2_ or that of CC-UA_2_, demonstrating the high purity of UA prodrugs.

### 3.2. Characterization of TK-NPs

**Figure 2.**
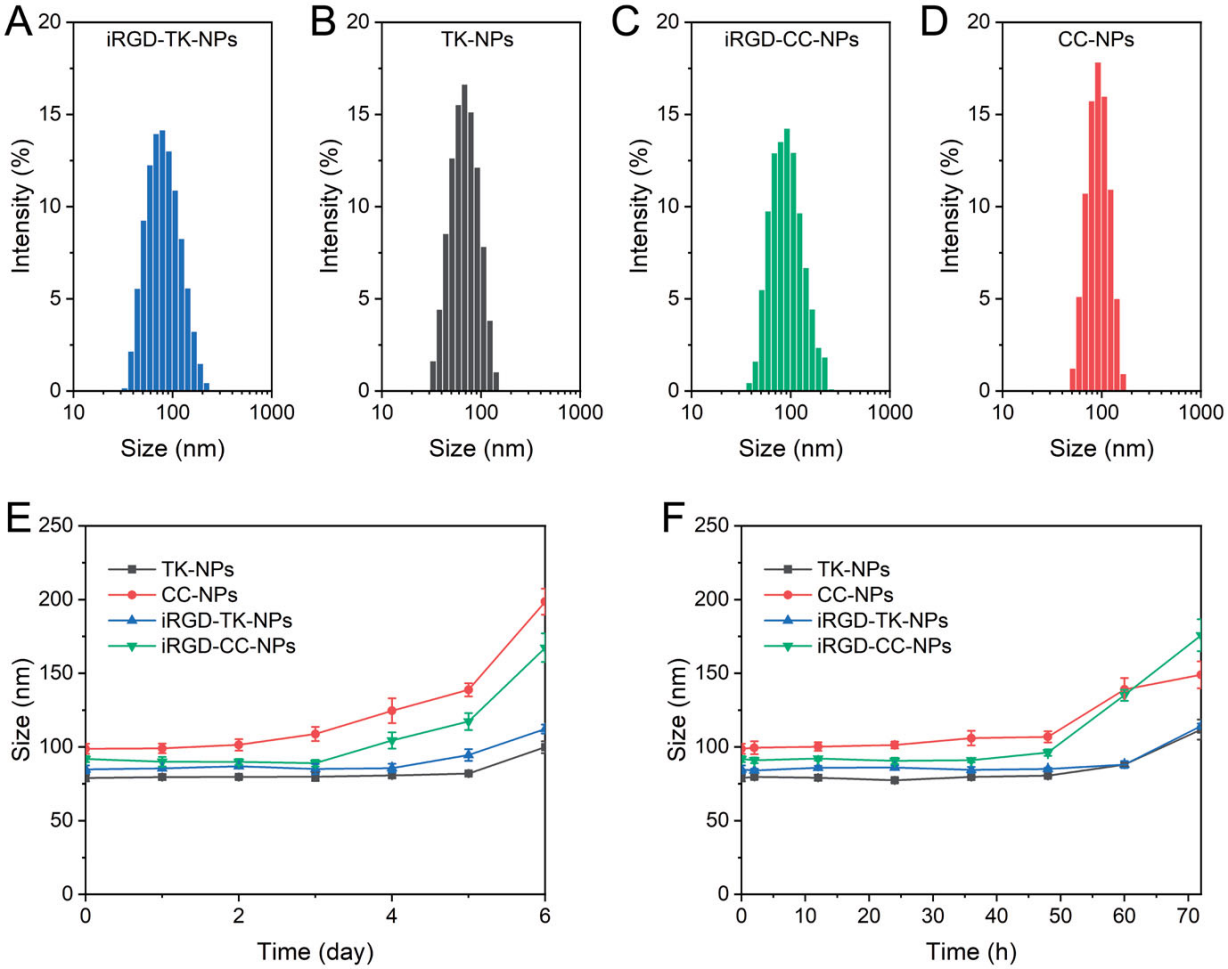
Hydrodynamic particle size of iRGD-TK-NPs (A), TK-NPs (B), iRGD-CC-NPs (C), and CC-NPs (D). (E, F) Size changes of iRGD-TK-NPs, TK-NPs, iRGD-CC-NPs, and CC-NPs in PBS at 4 °C (D) and PBS with 10% FBS (E) (*n*= 3).

According to our initial hypothesis, UA prodrugs may self-assemble into NPs in an aqueous solution without PEGylation. However, the size of both TK-UA_2_ and CC-UA_2_ NPs exceeded 400 nm, with poor colloidal stability. To solve this problem, lipid-PEG (DSPE-PEG) was incorporated. TK-UA_2_ and CC-UA_2_ can self-assemble with DSPE-PEG to form stable NPs. DLS was used to characterize the iRGD-TK-NPs, TK-NPs, CC-NPs, and iRGD-CC-NPs; the results are shown in Figure 2(A–D) and Table 1. The hydrodynamic size of iRGD-TK-NPs, TK-NPs, CC-NPs, and iRGD-CC-NPs was (84.8 ± 2.7), (78.9 ± 2.2), (98.8 ± 3.3), and (91.8 ± 2.1) nm, respectively. The polymer disperse index (PDI) value of iRGD-TK-NPs, TK-NPs, CC-NPs, and iRGD-CC-NPs was (0.15 ± 0.02), (0.23 ± 0.02), (0.24 ± 0.03), and (0.21 ± 0.03), indicating that these NPs have excellent uniformity. The DLC of iRGD-TK-NPs, TK-NPs, CC-NPs, and iRGD-CC-NPs reached 55.2%, 57.7%, 51.2%, and 50.1%, respectively, which was higher than that of traditional UA-based delivery systems (usually less than 10 wt%) (Ji et al., 2018; Shen et al., 2018). Moreover, these NPs had a negative surface charge, which may derive from PEG and the carboxyl group of UA. The negative surface charge of NPs was attributed to their strong resistance to nonspecific protein adsorption in blood circulation, resulting in prolonged circulation time (Yuan et al., 2012). To confirm the colloidal stability of four NPs, the variation in the sizes of these NPs in the simulated medium was measured at various time points. As shown in Figure 2(E), the diameters of four NPs had no significant changes after storage in PBS (pH 7.4) at 4 °C over five days, indicating the high stability of these NP types in these storage conditions. Additionally, iRGD-TK-NPs and TK-NPs retained a stable size in PBS with 10% FBS over 60 h, and iRGD-CC-NPs and CC-NPs maintained stability over 48 h (Figure 2(E)). These results confirmed the good colloidal stability of four NPs. The stability of iRGD-TK-NP and TK-NPs was higher than that of iRGD-CC-NPs as well as CC-NPs, which may be due to the fact that the TK linkage improves the structural flexibility of dimeric prodrugs, thereby increasing the stability of DPNS (Yang et al., 2020).

**Table 1.** Characterization of NPs.

NPs	Size (nm)	PDI	Zeta potential (mV)	DLC (%)	DEE (%)
iRGD-TK-NPs	84.8 ± 2.7	0.15 ± 0.02	–(16.8 ± 1.6)	55.2 ± 1.6	82.6 ± 2.7
TK-NPs	78.9 ± 2.2	0.23 ± 0.02	–(18.5 ± 2.6)	57.7 ± 2.3	83.7 ± 3.0
iRGD-CC-NPs	91.8 ± 2.1	0.21 ± 0.03	–(17.7 ± 0.7)	50.1 ± 2.1	75.3 ± 2.8
CC-NPs	98.8 ± 3.4	0.24 ± 0.03	–(18.6 ± 1.0)	51.2 ± 1.8	77.1 ± 2.6

### 3.3. Evaluation of ROS-sensitivity

**Figure 3.**
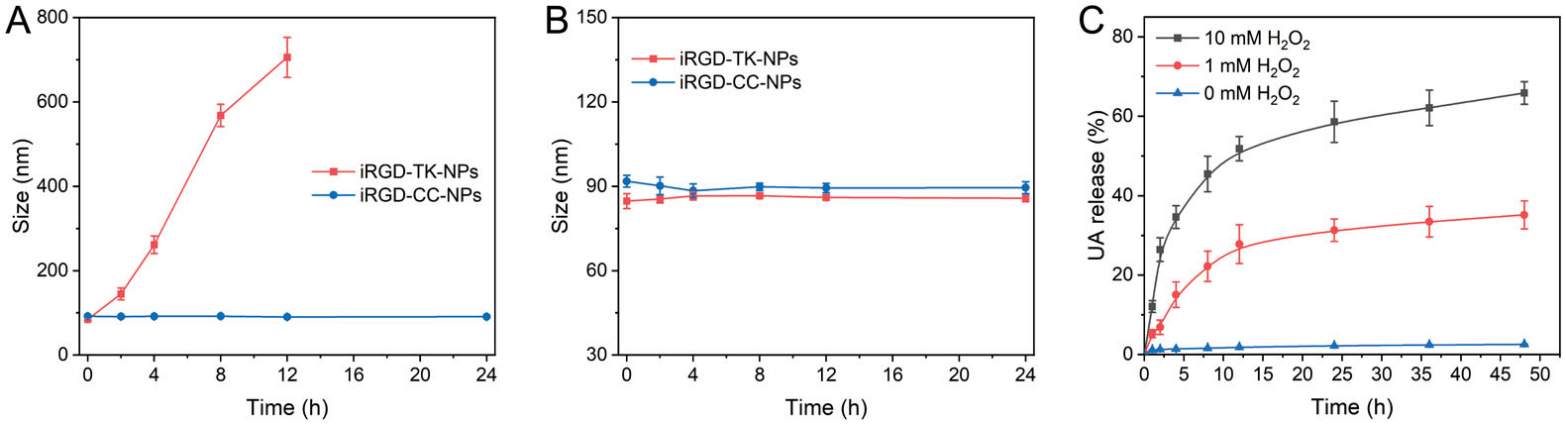
Evaluation of ROS-responsiveness. Size changes of iRGD-TK-NPs and iRGD-CC-NPs after incubation in PBS (pH 7.4) with (A) or without (B) 10.0 mM H_2_O_2_ (*n*= 3). (C) Drug release profiles of UA from iRGD-TK-NPs in the presence of 0, 1.0 mM H_2_O_2_, and 10.0 mM H_2_O_2_, respectively (*n*= 3).

Next, the sensitivity of NPs to ROS was investigated by evaluating the size changes and *in vitro* drug release of iRGD-TK-NPs and iRGD-CC-NPs under various ROS conditions. As shown in Figure 3(A,B), iRGD-CC-NPs maintained a stable particle size at pH 7.4 both with or without 10.0 mM H_2_O_2_, suggesting the ROS-insensitivity of iRGD-CC-NPs. Similarly, the size of iRGD-TK-NPs in PBS without H_2_O_2_ was also stable during the incubation time. However, the diameter of iRGD-TK-NPs changed from 85 to 145 nm and 706 nm after culturing in PBS containing 10.0 mM H_2_O_2_ for 2 h and 12 h, respectively, demonstrating the high ROS-responsiveness of iRGD-TK-NPs.

To further confirm ROS sensitivity, *in vitro* UA release behaviors of iRGD-TK-NPs were also evaluated, and the results are shown in Figure 3(C). It was found that the UA released from iRGD-TK-NPs showed H_2_O_2_-concentration-dependence. Negligible UA release from iRGD-TK-NPs occurred in the absence of ROS. When the addition of H_2_O_2_, UA release behavior was enhanced and sustained. In the presence of 1 mM H_2_O_2_, around 25% of UA was released from NPs after 48 h incubation. Interestingly, NPs underwent rapid release within 6 h under 10.0 mM H_2_O_2_, with about 65% UA release after incubation for 48 h. These results further confirm the high ROS sensitivity of iRGD-TK-NPs, which could promote the rapid conversion of the UA prodrug into the active parent drug, and ultimately enhance the antitumor effect of UA.

### 3.4. Cellular uptake

**Figure 4.**
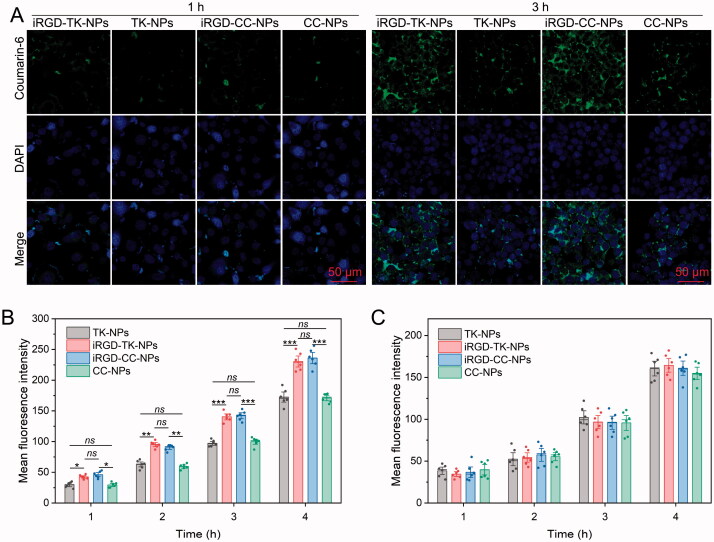
Cell uptake evaluation. (A) CLSM images of SGC 7901 cells after incubation with coumarin-6 loaded iRGD-TK-NPs, TK-NPs, iRGD-CC-NPs, and CC-NPs for 1 h and 3 h, respectively. FCM results of SGC 7901 (B) and NIH-3T3 (C) cells after treated with coumarin-6 loaded iRGD-TK-NPs, TK-NPs, iRGD-CC-NPs, and CC-NPs for 1, 2, 3, or 4 h, respectively (*n*= 6); *p< .05, **p< .01, ***p< .001, *ns*: no significance.

After reaching tumor tissue, the absence of cell-specific interaction between NPs and cells could induce insufficient cell uptake of NPs and drug expulsion, ultimately decreasing the antitumor effect, and even induce multidrug resistance (Wei et al., 2017; Yang et al., 2018; Mahmoudi Saber, 2019). The use of surface-modified targeting ligands is an effective strategy to overcome this problem. Herein, we incorporated iRGD into UA-based DPNS to increase its tumor-targeting ability. CLSM and FCM were performed to evaluate the ability of iRGD-TK-NPs to target cancer cells using the human GC SGC 7901 cell line (a cell line that has been reported to highly overexpress α_v_β_3_ integrins) and the normal NIH-3T3 cell line (Shi et al., 2019). In the CLSM images of both cells (Figure 4(A)), the intracellular concentration of iRGD-modified NPs (iRGD-TK-NPs and iRGD-CC-NPs) and no-iRGD-modified NPs (TK-NPs and CC-NPs) was increased from 1 h to 3 h, evidenced by the enhancement in the green fluorescence signal of coumarin-6 with prolonged incubation time, indicating that four NPs could effectively deliver UA into cancer cells. However, at the same time interval, the green fluorescence intensity in iRGD-modified NPs-treated SGC 7901 cells was remarkably higher than that of without iRGD-modified NPs. Additionally, no significant difference was observed between iRGD-TK-NPs and iRGD-CC-NPs in SGC 7901 cells. The fluorescence in SGC 7901 cells between TK-NPs and CC-NPs was also no remarkable difference. These results were also quantitatively verified by FCM. As shown in Figure 4(B), the mean fluorescence intensity (MFI) in SGC-7901 cells incubated with iRGD-TK-NPs and iRGD-CC-NPs for 1, 2, 3, and 4 h was 1.1-/1.3-fold, 1.2-/1.4-fold, 1.3-/1.5-fold, and 1.5-/1.6-fold higher than in the TK-NPs-treated group, respectively. The MFI in SGC 7901 cells incubated with iRGD-TK-NPs and iRGD-CC-NPs for 1, 2, 3, and 4 h was 1.4-/1.6-fold, 1.6-/1.5-fold, 1.4-/1.4-fold, and 1.3-/1.4-fold higher than in the CC-NPs-treated group, respectively. Moreover, MFI of coumarin-6 in the normal NIH-3T3 cells (Figure 4(C)) after culturing with NPs coated with or without iRGD showed no remarkable difference over the same period. These results suggest that NPs modified with iRGD can be specifically and selectively internalized by cancer cells through iRGD-mediated internalization.

### 3.5. iRGD-TK-NPs selectively enhance cytotoxicity

**Figure 5.**
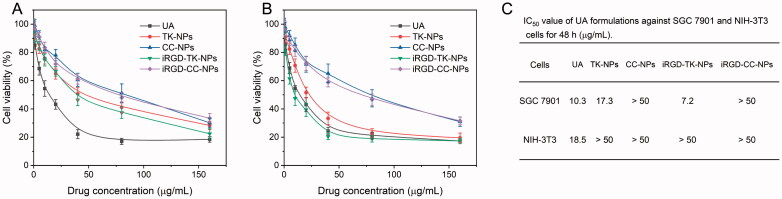
Cytotoxicity evaluation. Cell relative viability of NIH-3T3 cells (A) and SGC 7901 cells (B) after treatment with iRGD-TK-NPs, TK-NPs, CC-NPs, and iRGD-CC-NPs for 48 h, respectively (*n*= 6). (C) IC_50_ values of iRGD-TK-NPs, TK-NPs, CC-NPs, and iRGD-CC-NPs against SGC 7901 and NIH-3T3 cells for 48 h.

*In vitro* cytotoxicity assayed by the MTT method against SGC 7901 and NIH-3T3 cells were used to validate whether the iRGD-TK-NPs could effectively suppress the growth of cancer cells. The relative cell viability and IC_50_ value of all formulations are shown in Figure 5. In normal NIH-3T3 cells (Figure 5(A)), the four NPs showed slight suppression of cell proliferation (IC_50_ value >50 μg/mL, Figure 5(C)), caused by intracellular incomplete drug release, because of the low level of ROS in NIH-3T3 cells. For SGC 7901 cells, as presented in Figure 5(B), all formulations exhibited dose-dependent cell proliferation-inhibiting effects, and iRGD-TK-NPs showed higher cytotoxic activity. The IC_50_ value of iRGD-TK-NPs was 7.2 µg/mL, which was 1.4-fold, 2.4-fold, 2.4-fold, and 15.7-fold lower than that of free UA, TK-NPs, CC-NPs, and iRGD-CC-NPs, respectively (Figure 5(C)). The low cytotoxicity of free UA may mainly due to poor water solubility. The enhancement *in vitro* antitumor effect of iRGD-TK-NPs relative to TK-NPs was attributed to iRGD-mediated active targeting of cancer cells; this result was well consistent with the above cellular uptake results. Considering the cell internalization process of both iRGD-coated NPs, the lower cytotoxic activity of iRGD-CC-NPs was mainly due to intracellular slow and incomplete UA release. This phenomenon was also observed between TK-NPs and CC-NPs.

### 3.6. Pharmacokinetics and distribution

**Figure 6.**
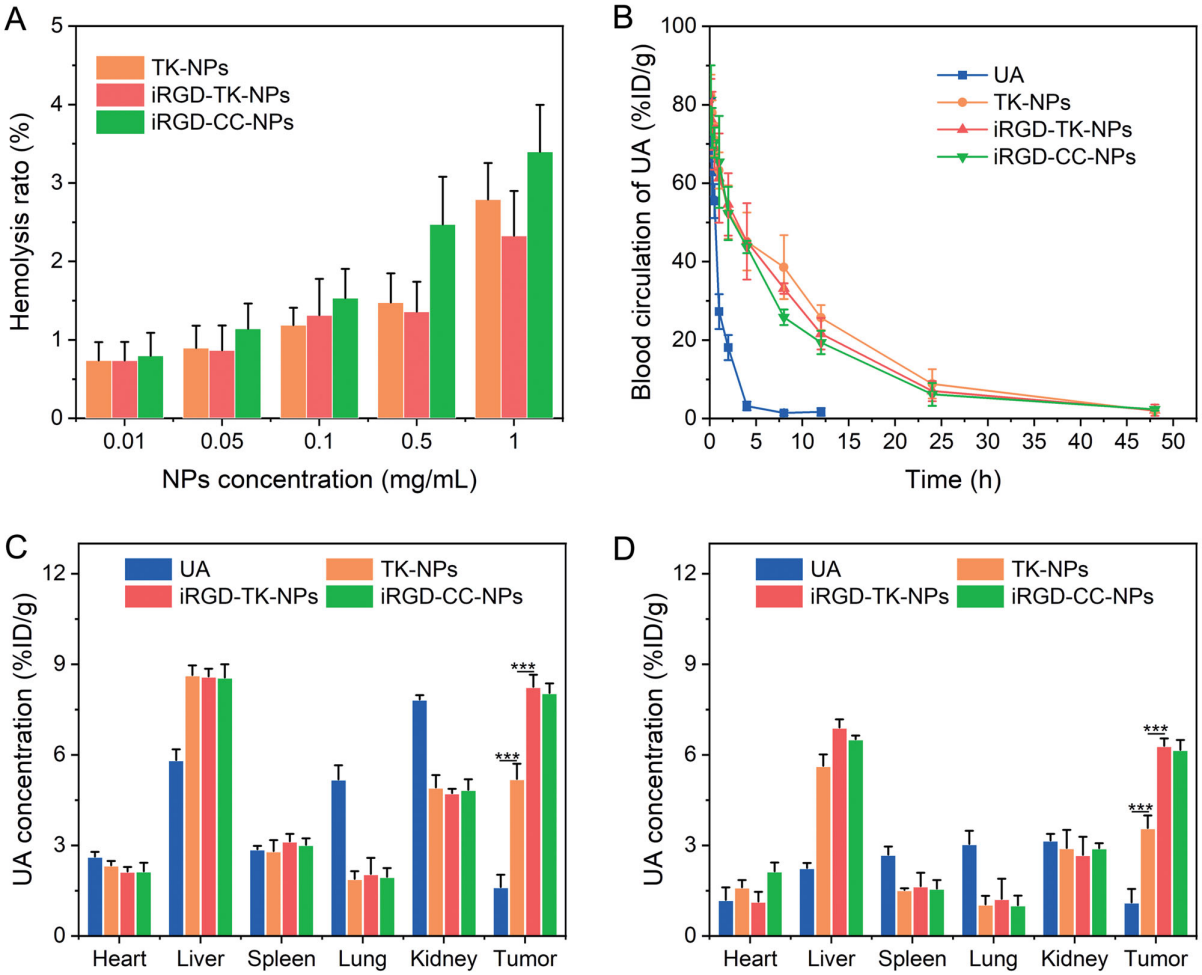
(A) Hemolysis ratio of TK-NPs, iRGD-TK-NPs, and iRGD-CC-NPs (*n*= 3). (B) Plasma concentration of TK-NPs, iRGD-TK-NPs, and iRGD-CC-NPs in SD rats at various times after a single intravenous injection (*n*= 6). The concentration of UA and the prodrug in major organs and tumor tissue of SGC 7901 tumor-bearing mice after intravenous injection with TK-NPs, iRGD-TK-NPs, and iRGD-CC-NPs for 6 h (C) and 12 h (D), respectively, (*n*= 6).

As the NPs designed and prepared here are administered via intravenous injection, their biocompatibility in the blood is very important. Therefore, the hemolytic analysis was performed before *in vivo* study to evaluate biocompatibility. As shown in Figure 6(A), all NPs showed good blood compatibility with a hemolysis ratio of lower than 5% at the UA concentration range of 0.01–1 mg/mL.

Subsequently, the pharmacokinetics of all UA formulations were investigated using the SD rat as the tumor model, and the results are shown in Figure 6(B). UA was rapidly cleared from the blood, and no drug could be detected after administration for 12 h. In contrast, all NPs significantly increased the retention time of UA in blood, and about 7.0, 8.9, and 6.2 µg/mL of UA iRGD-TK-NPs, TK-NPs, and iRGD-CC-NPs, respectively, in blood at 24 h post-injection. The area under the curve (AUC) of UA in iRGD-TK-NPs, TK-NPs, and iRGD-CC-NPs was 7.4-fold, 7.8-fold, and 6.8-fold higher than that of free UA, respectively. It is worth noting that the pharmacokinetic behavior of iRGD-TK-NPs, TK-NPs, and iRGD-CC-NPs did not significantly differ, indicating that the iRGD modification on the surface of NPs and difference in linkages between the two drug molecules did not remarkably influence the pharmacokinetic behavior of the prodrugs. The prolonged blood circulation could be attributed to NPs aggregating in tumor tissue through enhanced permeability and retention (EPR) effect-mediated passive-targeting.

We next evaluated the biodistribution of all UA formulations in SGC 7901 tumor-bearing mice. After a single intravenous injection at the dose of 11 mg/kg (equal to UA), the UA and UA-prodrugs in the major organs and tumor tissues at 6 h and 24 h postinjection were measured by HPLC. As described in Figure 6(C,D), after administration, free UA mainly accumulated in the liver and kidney and was cleared as time passed. The accumulated amount of free UA in tumor tissue was significantly lower than that of TK-NPs, iRGD-TK-NPs, and iRGD-CC-NPs both at 6 h and 12 h after administration. The total amount of free UA in tumor tissue was 3.2-/3.2-fold, 5.1-/5.7-fold, and 5.0-/5.6-fold lower than that of TK-NPs, iRGD-TK-NPs, and iRGD-CC-NPs post-injection for 6 h and 24 h, respectively. High levels of tumor accumulation of TK-NPs are mainly attributed to ERP effect-mediated tumor passive targeting. As expect, iRGD-coated NPs showed high levels of tumor targeting, and the concentration of iRGD-TK-NPs and iRGD-CC-NPs in tumor tissue was 1.6-/1.5-times and 1.8-/1.7-times higher than that of TK-NPs at 6 h and 12 h post-injection, respectively. This is attributed to iRGD-mediated active targeting and EPR effect-mediated passive targeting. Moreover, the concentration of iRGD-TK-NPs and iRGD-CC-NPs in tumor tissue was not significantly different in the same period.

### 3.7. *In vivo* antitumor effect

**Figure 7.**
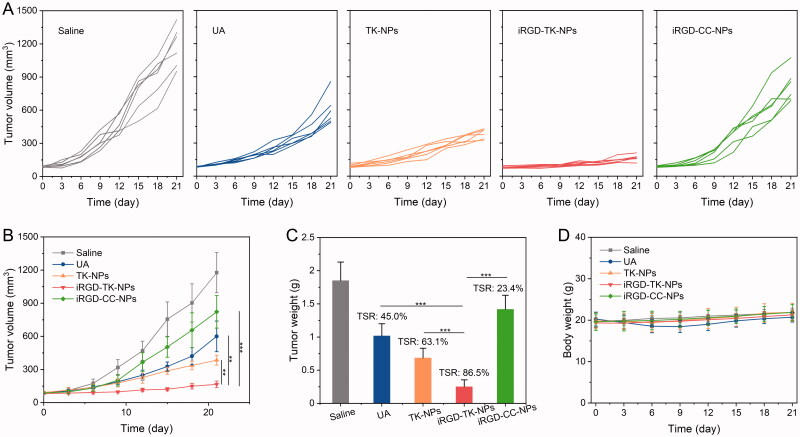
*In vivo* therapeutics of UA formulations. (A, B) Tumor volume changes of SGC 7901 xenografted mice after treatment with saline, UA, TK-NPs, iRGD-TK-NPs, and iRGD-CC-NPs (*n*= 6). (C) Tumor weight and tumor suppression ratio of SGC 7901 xenografted mice after treatment with saline, UA, TK-NPs, iRGD-TK-NPs, and iRGD-CC-NPs (*n*= 6). (D) Bodyweight changes in mice after therapy with saline, UA, TK-NPs, iRGD-TK-NPs, and iRGD-CC-NPs (*n*= 6).

Finally, mice bearing SGC 7901 tumors were used as animal models to investigate the *in vivo* antitumor effect of all UA formulations. The profile of changes in tumor volume, tumor weights at day 21, and TSR is shown in Figure 7(A–C). Compared with saline, free UA elicited moderate tumor inhibition and TSR only 45.0%. The poor *in vivo* antitumor effect of free UA is attributed to its poor solubility, short blood circulation time, and low accumulation in tumor tissue. In contrast, iRGD-TK-NPs showed the best out of antitumor effect, and its TSR was 86.5%, which was 1.4-fold and 3.7-fold higher than that of TK-NPs and iRGD-CC-NPs, respectively. The good antitumor effect of iRGD-TK-NPs is attributable to specific tumor-targeting and intracellular rapid and complete drug release. Additionally, body weights of mice in all formulation-treatment groups had no significant decrease (Figure 7(D)), indicating that these UA formulations show good biocompatibility.

## 4. Conclusions

In summary, a novel and simple ROS-responsive UA-based DPSN (iRGD-TK-NPs) was rationally designed and prepared in this study, and shown to exhibit significantly improved solubility and prolong the blood circulation time of UA. IRGD-TK-NPs have suitable zeta potentials, appropriate particle sizes, uniform spherical shape, high drug loading, and excellent biostability. Furthermore, IRGD-TK-NPs can significantly extend blood retention time of UA, specifically recognize and bind to α_v_β_3_-overexpressing GC cells guided by iRGD, effectively deliver UA into cancer cells, and selectively and rapidly release the drug intracellularly. The enhanced antitumor effect of iRGD-TK-NPs has been demonstrated against GC cells and GC-bearing mice. All experimental data confirmed that the UA-based ROS-sensitive DPSN could dramatically improve the anti-GC-cancer efficiency of UA. Therefore, the novel approach described herein may promote the clinical application of UA.
